# Atraumatic Deltoid Rupture with a Chronic Massive Rotator Cuff Tear: A Case Report and Surgical Technique

**DOI:** 10.1155/2022/1833988

**Published:** 2022-04-30

**Authors:** Matthew G. Alben, Neil Gambhir, Michael A. Boin, Mandeep S. Virk, Young W. Kwon

**Affiliations:** Division of Shoulder and Elbow Surgery, Department of Orthopedic Surgery, NYU Grossman School of Medicine, NYU Langone Orthopedic Hospital, NYU Langone Health, New York, NY, USA

## Abstract

**Case:**

We report a rare case of a spontaneous, atraumatic rupture of the anterior and middle heads of the deltoid with an underlying massive rotator cuff tear. Unique clinical findings included a palpable mass of torn deltoid distally with a proximal tissue defect. Magnetic resonance imaging of the deltoid demonstrated complete tear of the anterior head; involvement of the middle head was found intraoperatively. Given the acute nature of injury and potential impact on the feasibility of future reverse shoulder arthroplasty, surgical repair of the torn deltoid was discussed with the patient and performed via superior approach.

**Conclusion:**

Direct surgical repair is a viable treatment option if diagnosed early.

## 1. Introduction

Rupture of the deltoid muscle is a rare condition that can result in disabling functional limitations of the arm. It is most often reported in literature as a postoperative complication after open rotator cuff repair (RCR) or reverse total shoulder arthroplasty (rTSA) where the anterior deltoid was released for surgical exposure. Glenohumeral steroid injections, chronic rotator cuff tears (RCTs), and trauma have all also been described as potential predisposing etiological factors [1–6]. In cases with concomitant RCTs, early recognition and treatment is crucial as management options can become drastically limited [7].

We report a rare case of spontaneous atraumatic deltoid rupture with an underlying massive RCT. Herein, we describe the unique clinical presentation, advanced imaging findings, intraoperative findings, and 12-month postoperative outcome. The patient provided consent for data concerning the case to be submitted for publication.

## 2. History and Preoperative Findings

**Figure 1:**
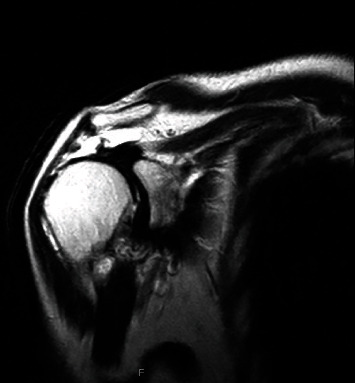
Coronal view of a T2-weighted MRI of the right shoulder following consultation for a rotator cuff tear.

The patient is a 73-year-old, right-handed female with a past medical history of a chronic massive RCT of the right shoulder diagnosed 3 years prior to current presentation. At that time, she described unremitting pain in the anterior shoulder exacerbated by overhead activities and lifting objects. Magnetic resonance imaging (MRI) confirmed a massive RCT with supraspinatus, infraspinatus, and subscapularis muscle atrophy (Figure 1). As her symptoms persisted despite a course of physical therapy, surgical treatment with rTSA was recommended. However, given reasonable functional capacity, she refused surgical intervention.

**Figure 2:**
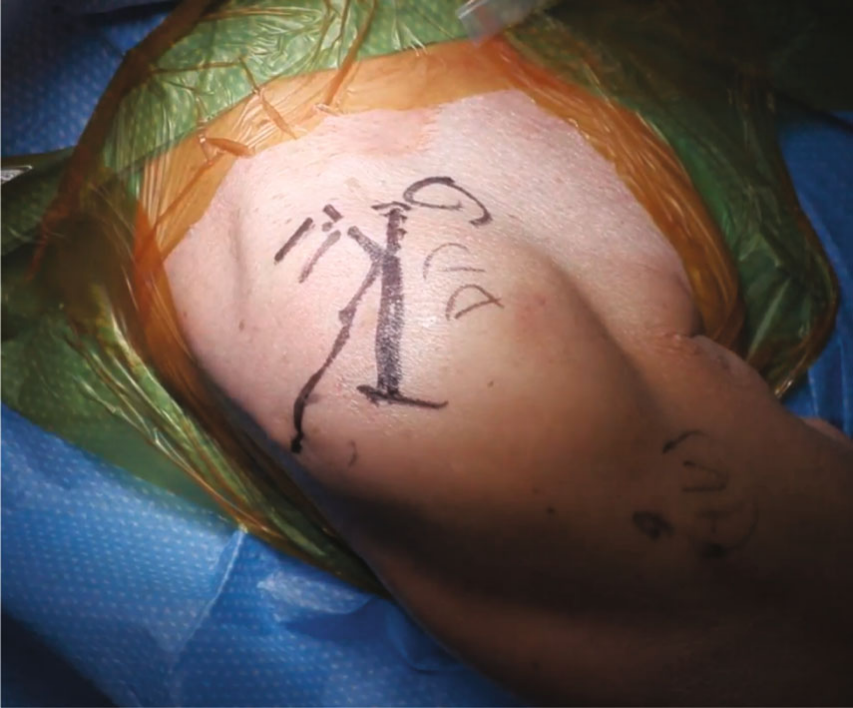
With the patient in a beach chair position, the defect of the torn right deltoid muscle is visible just distal to the antero-lateral aspect of the acromion.

**Figure 3:**
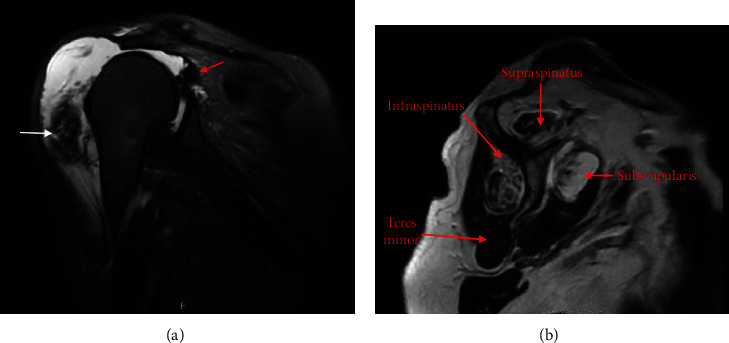
Coronal (a) and sagittal (b) view of a T2-weighted MRI of the right shoulder showing a large effusion with rupture of the deltoid (white arrow) and rotator cuff tear (red arrow).

Nine days prior to the current presentation, the patient noted an acute onset of weakness, pain, bruising, and deformity of the right shoulder. She denied any significant lifting, falls, or trauma that led to her symptoms despite multiple questions. Physical examination was remarkable for a palpable defect just distal to the antero-lateral aspect of the acromion (Figure 2). The torn deltoid muscle was retracted and rolled back distally resulting in a palpable mass. In addition to preexisting strength deficits related to massive RCT (external rotation lag sign, drop arm sign), there were deficits in abduction due to deltoid deficiency. Passive range of motion (ROM) of the right shoulder was severely limited by pain and guarding. A new MRI reconfirmed the massive RCT involving the supraspinatus, infraspinatus, and subscapularis tendons, all with fatty atrophy (grade 3 and higher) and anterior deltoid head rupture from the acromion with distal migration of the muscle mass (Figure 3).

Given the acute nature of the injury and potential impact on feasibility of future rTSA, extensive discussion was conducted regarding treatment options which included nonoperative treatments (observation and supervised therapy if or when pain improved), surgical repair of the deltoid only, or surgical repair of the deltoid with concomitant insertion of rTSA. After much consideration, the patient decided to proceed with the surgical repair of the deltoid without rTSA.

## 3. Surgical Treatment and Postoperative Care

**Figure 4:**
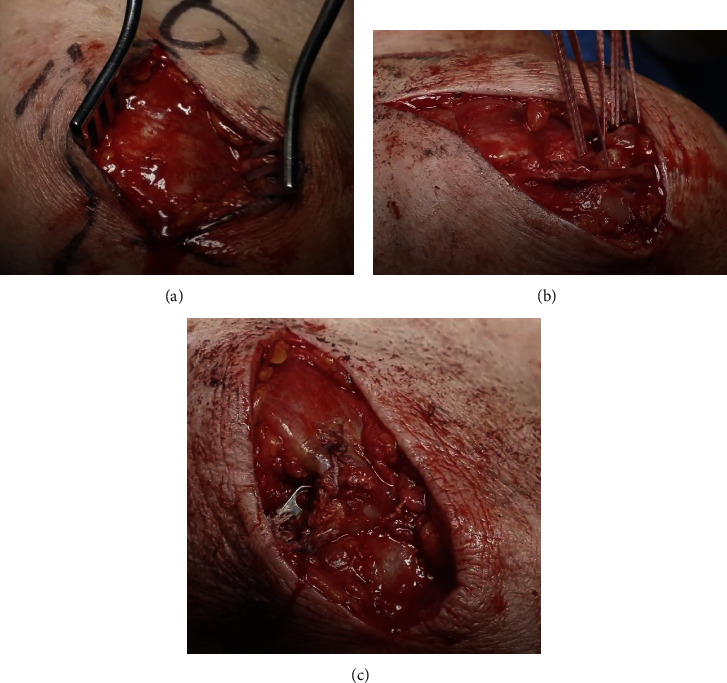
(a) Intact fascia directly overlying the torn right deltoid muscle. (b) Deltoid muscle secured by strong resorbable sutures (FiberTape sutures x3 [Arthrex, Naples, FL.]) passed in a Mason Allen configuration. (c) Deltoid repair after sutures were tied over a 3-hole mini plate (Stryker, Kalamazoo, MI) on the superior surface of the acromion.

With the patient in a beach chair position, a superior incision was made from the coracoid process, over the anterior lateral corner of the acromion, extending posteriorly by about 8 cm. Upon blunt subcutaneous tissue dissection, the thin fascia overlying the anterior and lateral portion of the deltoid was noted. In fact, the torn deltoid could not be immediately visualized as this fascia was directly overlying the muscle (Figure 4(a)). With palpation, however, the area of rupture could clearly be identified. The fascia was incised distally from the antero-lateral corner of the acromion. Residual hematoma was immediately identified and evacuated.

The underlying shoulder joint was evaluated and the massive RCT was confirmed and retracted medial to the glenoid rim. The tear included all of the supraspinatus and infraspinatus tendons, as well as the upper subscapularis tendon. Despite attempts, the tendons could not be mobilized for repair.

Intraoperative inspection of the deltoid revealed that both the anterior and middle deltoid heads had avulsed from their origin and retracted distally and posteriorly. The deltoid muscle was secured provisionally with strong resorbable sutures (FiberTape sutures x3 [Arthrex, Naples, FL.]) passed in a Mason Allen configuration as described by Gerber et al. (Figure 4(b)) [8]. After blunt mobilization for maximal excursion, the deltoid origin eventually could easily reach the acromion to restore normal anatomy. The sutures were passed vertically from the inferior to superior surface of the lateral and anterior border of acromion via drill holes. In order to minimize the likelihood of bone “cut out,” these sutures were tied over a 3-hole mini plate (Stryker, Kalamazoo, MI) on the superior surface of the acromion with the arm in abduction to reduce stress on the repair (Figure 4(c)). During movement of the shoulder, even at full adduction and rotation, minimal stress was noted at the repair site. The wound was then closed in layers.

The arm was immobilized in a splint with the shoulder in 30 degrees of abduction for the first 2 weeks, which was then switched to a simple sling. Physical therapy for passive motion was scheduled to start at 4 weeks with the allowance for active motion at 8 weeks.

## 4. Outcome

Due to COVID-19 pandemic, the patient was able to comply with postoperative rehabilitation for only the first 9 weeks. At that time, her ROM revealed passive shoulder flexion to 100° and external rotation (ER), with the arm at the side, to 30°. Active flexion and ER were 60° and 20°, respectively. She did a home exercise program thereafter. One-year follow-up was conducted via telemedicine as the patient did not wish to return for an in-person visit due to the ongoing pandemic. The patient was asked to complete seven patient reported outcome measures (Table 1). When asked about her postoperative shoulder function, she stated it was grossly unchanged from preoperative status. Additionally, the patient reported that her pain is now minimal and noted discomfort only at the extremes of her shoulder ROM and after strenuous use.

## 5. Discussion

Deltoid ruptures have been reported in the setting of chronic RCTs, steroid injections, sclerotic changes, open RCR or acromioplasty, trauma, calcific tendinopathy, and chronic subacromial bursitis [3–6]. Sclerotic changes of the humeral greater tuberosity can accelerate the rate at which attrition occurs by creating frictional forces on the undersurface of the deltoid [3]. Calcific tendinopathy and chronic subacromial subdeltoid bursitis are two additional conditions where degeneration and chronic local inflammation, respectively, can lead to deltoid rupture [3]. Local and systemic steroid exposure can weaken both the deltoid tendon and muscle belly itself [2–4, 6]. Misplaced subacromial injections directly into the deltoid itself further the risk for a weakened deltoid [2]. Of all these risk factors, the only factor noted in this presented case was massive RCT.

Without balanced stabilizing forces provided by the rotator cuff muscles, activation of the deltoid leads to proximal humeral translation [4, 9–12]. In turn, the superiorly migrated humeral head may then impinge the deltoid origin against the edge of the acromion leading to muscle fiber degeneration with increased risk for rupture [2–5]. As such, it has been proposed that underlying RCTs can disrupt the intrinsic shoulder dynamics and lead to an atraumatic rupture of the deltoid muscle [4, 5, 9, 12]. Despite these theoretical concerns, to our knowledge, only 7 atraumatic deltoid ruptures are confirmed in the literature [2–4, 11].

From available reports, new onset or sudden worsening of shoulder pain is the most common presenting symptom; further suspicion may be raised by swelling, ecchymosis, arm weakness, and decreased ROM [2, 5]. In conjunction with these typical findings, our patient presented with a palpable defect just distal to the antero-lateral aspect of the acromion which has been reported to be fairly rare, occurring in only 8.3% of the patients described by Ilaslan et al. [5]. Of the 24 patients reported in their study, all patients suffered from tears to the middle deltoid [5]. Though initial intraoperative inspection of the ruptured deltoid was difficult to discern as the overlying fascia remained intact despite the injury, our patient revealed rupture to both the anterior and middle heads of the deltoid.

The available literature on deltoid ruptures is relatively sparse, but does include reports of these ruptures in the setting of an existing RCT [2–4, 11]. Ilaslan et al. and Colak et al. reported a review based on imaging of this injury. The former found in 8,562 shoulder MRIs, only 24 patients (0.3%) had both injuries while the latter found this concurrence in only 46 of the 111,889 shoulder MRIs reviewed (0.04%) [3, 5]. The remaining 4 articles found 7 patients with concurrent injuries, further affirming the scarcity of this diagnosis [2–4, 11]. Tay and Collin described a case treated by rTSA with good functional outcomes [11]. Though this may be possible in a very specific patient subset, deltoid function is critical for rTSA and typically not recommended unless deltoid function is optimal [5, 9, 12–14]. A superimposed tear of the deltoid in nonsurgically managed RCT patients can drastically reduce their functional capacity. Thus, an extensive discussion must address the potential long term limitations that can remain without surgical management.

## 6. Conclusion

Despite RCTs being among the most common shoulder pathologies to occur, subsequent atraumatic deltoid muscle rupture is a very rare complication [15]. RCT patients that present with acute shoulder pain, deformity, or sudden decrease in shoulder function should raise concern for injury to the deltoid muscle. Early identification can allow for surgical treatment although a preexisting RCT may preclude attainment of optimal functionality after deltoid repair.

## Figures and Tables

**Table 1:** 

	Postoperative score
PROMIS Upper Extremity	24.8 ± 2.2
PROMIS Pain Interference	63.3 ± 2.2
PROMIS Pain Intensity	38.3 ± 3.1
PROMIS General Life Satisfaction	61.0 ± 2.8
American Shoulder and Elbow Surgeons Score	48.3
Constant Murley	27
Subjective shoulder value	30

PROMIS: Patient-Reported Outcomes Measurement Information System.

## Data Availability

No archived datasets were relevant to the preparation of this manuscript.

## Additional Points

The authors of this paper certify that they have no affiliations with or involvement in any organization or entity with any financial or nonfinancial interests pertinent to the subject matter discussed in this manuscript.
